# Xanthogranulomatous cystitis in a child

**DOI:** 10.1590/0100-3984.2015.0225

**Published:** 2017

**Authors:** Roberto César Teixeira Dantas, Ivo Lima Viana, Camila Soares Moreira de Sousa, Breno Braga Bastos, Carla Lorena Vasques Mendes de Miranda

**Affiliations:** 1 Medimagem, Teresina, PI, Brazil.; 2 UDI 24 horas, Teresina, PI, Brazil.

**Figure 1:**
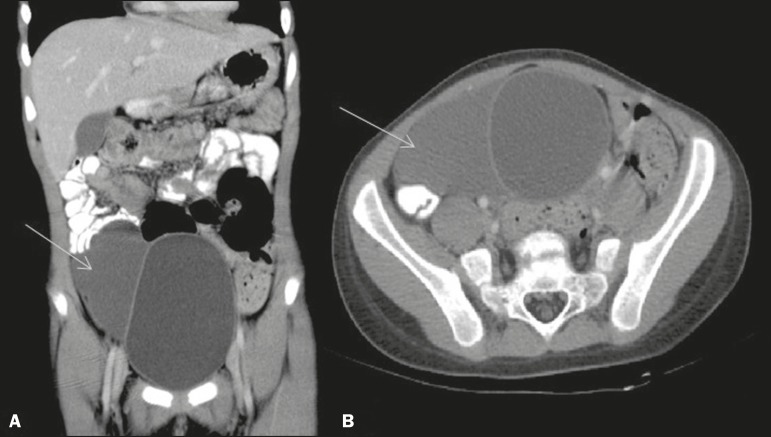
Coronal (**A**) and axial (**B**) reconstructions of contrast-enhanced computed tomography of the abdomen, showing a well-defined, hypointense collection with regular contours, with cystic attenuation, showing no enhancement and in contact with the right lateral wall of the bladder.

**Figure 2:**
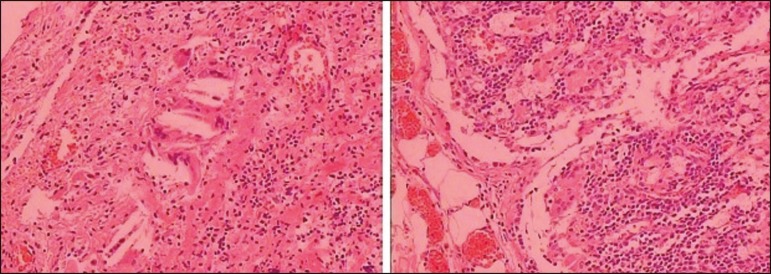
Histopathological section showing fibroadipose tissue with a xanthogranulomatous reaction (characterized by the presence of xanthomatous macrophages), a giant-cell reaction, cholesterol crystals, and mild chronic inflammatory infiltrate. A similar macrophage reaction can be seen in the lymph node.

Dear Editor,

A seven-year-old female patient with acute appendicitis underwent an emergency appendectomy. During the procedure, as incidental findings, a bulky bladder and a probable collection adhered to the wall were observed. Cystoscopy revealed an enlarged bladder with diffuse thickening of its walls. Subsequently, computed tomography of the abdomen showed a well-defined, hypointense collection, with cystic attenuation, with regular contours, showing no enhancement and in contact with the right lateral wall of the bladder (Figure 1). An investigation of pathological antecedents revealed that the patient had experienced recurrent episodes of cystitis in the last year. The decision was made to perform laparoscopic surgery, during which a small communicating orifice was identified (between the lesion and the interior of the bladder) and partial cystectomy was performed. Histopathological analysis demonstrated fibroadipose tissue exhibiting a xanthogranulomatous reaction (characterized by the presence of xanthomatous macrophages), together with a giant-cell reaction, cholesterol crystals, and mild chronic inflammatory infiltrate. A similar macrophage reaction was observed in the lymph node (Figure 2). In view of those findings, the main diagnostic hypothesis was xanthogranulomatous cystitis.

Xanthogranulomatous cystitis is a rare chronic inflammatory disease, only approximately 30 cases having been documented in the literature. It has a benign course and its origin remains obscure. However, previous reports have suggested possible associations with a remnant of the urachus, chronic infection, malignant bladder tumor, and immune disorders^(1)^. The clinical symptoms are non-specific and therefore do not facilitate the differential diagnosis with other diseases of the bladder. The most common forms of presentation are irritative urinary symptoms, a palpable mass in the abdomen, and hematuria^(2-4)^. Among the cases published in the literature, that the mean age at onset is approximately 46 years, with no gender predominance, and the preferential location is in the dome of the bladder^(1,4,5)^. However, the case presented here was in a seven-year-old (pediatric) patient, in whom the lesion was located in the right lateral wall, thus ruling out any association with the urachus.

In individuals with xanthogranulomatous cystitis, conservative treatment is not effective. Such individuals require surgical resection by partial cystectomy, which is currently the gold standard treatment fore the disease^(2-5)^. Xanthogranulomatous lesions can occur at sites other than the bladder, typically the kidneys or, less frequently, the gall bladder, pancreas, appendix, colon, ovary, endometrium, and brain, usually mimicking malignancy^(2-4)^.

Xanthogranulomatous cystitis is an extremely rare disease and continues to be the subject of many studies, because little is known about its true cause and behavior over the long term. This case highlights the importance of recognizing an unusual lesion that can present in individuals of any age and can impede the final diagnosis.
